# From political to democratic sophistication—A critical reappraisal

**DOI:** 10.1111/bjso.70035

**Published:** 2025-12-17

**Authors:** Klara Steinmetz, Frank Asbrock

**Affiliations:** ^1^ Chemnitz University of Technology Chemnitz Germany

**Keywords:** contradictions, interest in politics, political debate, political knowledge, political participation, political sophistication, political sophistication bias, tolerance of ambiguity

## Abstract

Political sophistication (PS) describes how individuals absorb political information from their environment, integrate it into existing knowledge structures and form political opinions accordingly. This paper explores the origins and development of PS, offers a critical classification and proposes new avenues for conceptualization. Drawing on the literature from psychology, political science and educational research, the paper traces the evolution of the concept and highlights a shift in how PS is understood. By reviewing research on bias and values, especially work that engages with PS, the analysis identifies both the lack of a coherent theoretical framework and inconsistencies in its measurement. Existing findings, such as the so‐called political sophistication bias, are critically re‐examined in this context. Building on this foundation, the paper introduces democratic sophistication as a refined conceptualization of PS, framing it as an individual‐level variable relevant to democratic citizenship. The potential of democratic sophistication is then explored concerning its role in social psychological research, its interdisciplinary relevance and its practical implications for understanding political discussion behaviour, mitigating polarization and fostering citizens' capacity for deliberative engagement.

The truth is rarely pure and never simple.

Oscar Wilde, The Importance of Being Earnest (1895)

Although nuanced political discourse that incorporates diverse viewpoints and perspectives constitutes a core element of democratic systems (Lipset, 1959; Pateman, 1970), such discourse has unfortunately been on the decline. The last few decades have been marked by numerous turbulent political events that illustrate a tendency towards polarization: Roe v. Wade, the Middle East conflict, and the recognition and management of climate change. This development emerges, among other factors, from the rising influence of identity politics, which has the potential to produce (affective) polarization and reduce complex political debates to oversimplified binaries, thereby endangering nuanced democratic discourse (Bonikowski et al., 2019; Milačić, 2021). But to do justice to the complexity of our world and the diverse lived realities of people, it requires a nuanced discourse that considers various perspectives and arguments, allowing for the endurance of contradictions rather than reducing debates to absolutes. While we need to foster such a kind of debate culture as a society, citizens must engage in political discourse and be active members of a democratic society. People who are especially interested in politics, are knowledgeable in this field and hold complex political attitudes that are coherent with their values, are sometimes referred to as politically sophisticated (Luskin, 1990). While this concept seems to be of interest for democratic education and political psychology, it is mostly discussed in political science literature. Research on political sophistication (PS) goes back to the 1960s (Converse, 2006) and includes perspectives on where it stems from and how it is related to attitudes (e.g. Luskin, 1987), values (e.g., Goren, 2004) and bias (Bakker et al., 2021). Unfortunately, there is a notable lack of coherence in the literature, in the way it is theoretically conceptualized as well as in how it is measured. This paper aimed to give an overview of the origins of PS, how the understanding of it developed over time and most importantly, how the lack of coherence leads to several erroneous assumptions, as the Political Sophistication Bias (e.g., Vitriol et al., 2023). As a result of my criticism, we introduce the idea of Democratic Sophistication, a concept focusing on individual‐level participation in a democratic system, to offer a more narrow and social psychological perspective on PS and illustrate how and why the construct still holds conceptual and practical value.

## WHAT IS POLITICAL SOPHISTICATION?

PS originates from political science research in the 1960s (Converse, 2006). This *traditional* approach (Gallina, 2023) examines the political belief system (PBS), in which political content and attitudes are organized in a cognitive network (Converse, 2006; Luskin, 1987, 1990). With political opinions and decisions depending on the network's structure (Sniderman et al., 1991; Zaller, 1992), people with a particularly differentiated and well‐developed belief system are politically sophisticated (Converse, 2006; Luskin, 1987). Such a PBS can be considered stable, grounded in core principles, and a well‐organized ideological framework, reflecting high political knowledge and cognitive engagement. In sum, PS gives us an idea of how people think about their political environment and come up with political attitudes.

PS has been frequently used as an individual‐level indicator for explaining voting behaviour (Bovan, 2022; Kölln, 2018), political intolerance (Gibson et al., 2020) and resistance to fake news (Vegetti & Mancosu, 2020; Weitz‐Shapiro & Winters, 2017). The broader aim of this research is to understand how people come up with their political beliefs that eventually lead to political actions (Krosnick et al., 2010; Reese & Jacob, 2015; Van Zomeren, 2013). While some social psychological studies have picked up the concept of PS (Carriere et al., 2019; Vitriol et al., 2023), most researchers focus on (ideological) attitudes (Beierlein et al., 2015; Duckitt & Sibley, 2016) or partisanship (Bolsen et al., 2014; Van Bavel & Pereira, 2018) to explain political behaviour. Nevertheless, PS might have potential in explaining political attitudes and behaviour and deserves interdisciplinary consideration, as we will elaborate.

The focus of PS research over the years shifted from examining how people think about their political environment to what people know about politics. Specifically, in past years, researchers focused on how political knowledge is related to support for specific parties, politicians and policy issues (Sherkat, 2021; Van Der Heijden & Verkuyten, 2020). In order to shed light on this paradigm shift, Gallina (2023) introduced a classification centred on cognitive and pragmatic sophistication. The cognitive and rather traditional approach to PS (e.g., Vitriol et al., 2023) examines how people think about politics, while the pragmatic approach (e.g., Zingher & Flynn, 2019) focuses on what people know about politics. These categories, however, are applied retrospectively to organize the literature on PS, which means that they are not inherent in its research. Cognitive understandings of PS are often conflated with pragmatic approaches to its measurement. While this categorization may be useful, it cannot resolve the lack of theoretical clarity and coherence within the field. Nevertheless, we will refer to these distinctions throughout the paper to trace the conceptual development of PS. Additionally, we will draw on research that links PS to values and bias, as well as social psychology approaches, to highlight conceptual weaknesses and offer critical reflections.

### The origins of political sophistication research: Political belief system networks

In the first half of the 20th century, researchers examined citizens' political knowledge, reaching sobering conclusions (Berelson et al., 1955; Downs, 1957; Hyman & Sheatsley, 1947; Lippmann, 1922). In 1960, the Michigan school aimed to categorize individuals by political expertise (Campbell et al., 1960), based on the use of abstract ideological categories and the differentiation and correctness of political thought. This work was later continued in the examination of political belief systems (Converse, 2006), seen as cognitive networks that help us comprehend our political surroundings and make sense of new information. They hold contents and attitudes which are more or less central, and therefore stable, to a person. High amounts of constraints are interpreted as a consequence of organization, which helps people to make sense of new information. Accordingly, someone with a broad and well‐organized belief system should be able to process and understand new information more easily and can, therefore, be considered more politically sophisticated.

Even though Converse (2006) did not clearly define the construct of PS, he coined the term as a state of a highly differentiated and broadly developed PBS. The understanding of abstract categories, such as liberal and conservative, was used to operationalize the state of their political belief system through recognition, description and usage of these terms (Converse, 2006). Based on that, people were categorized into different groups. Besides their expertise in these abstract categories, the groups also differed in terms of their political information, education and political involvement; in sum, in their political PS. Additionally, those who were highly sophisticated disclosed a higher relation between their jobs and their voting behaviour, identified as loyal partisans and had stable political attitudes.

While belief systems include certain attitudes, being politically sophisticated is not necessarily connected to a specific content alignment of ideology but simply describes the ability of people to make sense of their political environment. Converse uses the term ideology, though, to explain that highly sophisticated individuals are more stable and consistent in their political attitudes, while those less sophisticated have more fragmented political opinions, leading to more electoral volatility (Dassonneville & Dejaeghere, 2014). That said, Converse denotes highly sophisticated as highly ideological but does not associate a specific ideology. The term PS can, therefore, rather be understood as an individual skillset that helps to understand the political landscape, how society works and how different political topics are connected, as the following quote illustrates:

Most members of the mass public, however, fail to proceed so far. Certain rather concrete issues may capture their respective individual attentions and lead to some politically relevant opinion formation. This engagement of attention remains narrow, however: Other issues that any sophisticated observer would see as “ideologically” related to the initial concern tend not to be thus associated in any breadth or number. The common citizen fails to develop a more global point of view about politics. A realistic picture of political belief systems in the mass public, then, does not omit issues and policy demands completely nor one that presumes widespread ideological coherence; it is rather one that captures with some fidelity the fragmentation, narrowness and diversity of these demands. (Converse, 2006, p. 54)

While this approach to PS offers valuable insights into how people understand and evaluate their political environment, it has notable weaknesses. From the outset, there is an idea, without theoretical derivation or argumentation, of who is particularly politically sophisticated: career politicians (Converse, 2006, p. 2), men (Converse, 2006, p. 37) and people with formal education (Converse, 2006, p. 15). While denying women their own political opinions might simply be a representation of the 1960 misogynistic Zeitgeist, these assumptions are limited by claiming qualitative differences in people's cognitive systems, for which there is no indication of (Stanovich et al., 2019). Apart from the fact that there is no theoretical derivation of how education should affect PS, some studies restrict this relation (Highton, 2009; Luskin, 1990). Nevertheless, Converse assumes that people who have a higher social status are more sophisticated. This disregards the fact that people who suffer from an unequal distribution of resources, in particular, feel the pressure to be and act politically, which gives them a different approach to political discourse.

Furthermore, we criticize the misleading use of the term ‘ideology’. PBS research refers to politically sophisticated individuals having a stable set of political attitudes, describing them as ‘ideologues’ (Converse, 2006; Luskin, 1990).

According to Jost et al. (2009), an ideology is a “set of beliefs about the proper order of society and how it can be achieved” (p. 309). Here, ideologies are individual political belief structures with normative character, structural coherence, social sharing and intra‐individual stability. While this definition is consistent with the conceptualization of a highly sophisticated belief system as a tightly woven network of information that ensures the formation of stable political opinions almost intuitively, the utilization of this term in this regard is misleading. It suggests that highly sophisticated individuals use ideologies more than others to simplify their environment and make decisions. While it is indeed easier for highly sophisticated individuals to form opinions “online” based on a well‐developed belief system, it would be wrong to equate the application of a well‐developed PBS with the dogmatic application of an ideology that does not initially require any form of political expertise (Ball et al., 2014). Additionally, PS should not be associated with any specific ideological content or political orientation.

### From “How we think” to “What we know”

Based on the Michigan school approach, different lines of research have developed, each prioritizing a distinct aspect of PS. During the 1960s and 1970s, using people's understanding of ideological terms as a PS indicator was popular (Field & Anderson, 1969; Nie et al., 1976; Pierce, 1970) but faced criticism for methodological flaws (Luskin, 1987). Another line of research focused on the consistency of political opinions across different topics, assuming higher consistency in politically sophisticated individuals (Barton & Parsons, 1977; Weissberg, 1976). While the consistency of opinions was examined as an expression of PS, PS was later used to explain the consistent translation of values into political opinions (Carpini & Keeter, 1996; Goren, 2004; Sniderman et al., 1991; Zaller, 1992).

However, the focus was soon brought back to examining how people organize their political world, influenced by the 1980s schema theory. A schema refers to a mental framework that individuals use to organize and interpret information related to political beliefs, behaviours and attitudes (Lau, 1986), impacting how people perceive and engage with political issues (Marcus et al., 2000) as well as behaviour (Lodge & Taber, 2013). Though understanding PS as a complex and abstract cognitive system (Conover & Feldman, 1984) is close to the traditional approach of PBS research, the focus shifted towards a rather pragmatic understanding of PS: Instead of investigating *how people think about politics*, researchers started to look into *what people know about politics*, since a complex and well‐organized schema is supposed to result in knowledge (Luskin, 1987). PS, therefore, became synonymous with political expertise or factual knowledge. Instead of examining PS as how people think about politics, understanding it as what people know about politics became more prominent (Gallina, 2023).

Though this development could be referred to as a paradigm shift (Gallina, 2023), there is no shift in the conceptualization but only in the measurement of PS. Though measuring political knowledge is an efficient way to measure broad and complex constructs, it fails to capture PS's original meaning (Lupia & McCubbins, 1999, 2000; Popkin, 1995). Especially since we always have access to large amounts of information in the digital age, it seems all the more important to be able to classify and verify the validity of information. Besides, research shows that knowledge per se doesn't make people more competent in political discussions since they still call upon personal experiences rather than arguing rationally (Cramer & Toff, 2017). Additionally, if we measure political knowledge, we have to ask ourselves which level of factual knowledge can even be expected from citizens. What do we consider average?

### Explaining political sophistication

Although many researchers examined people's PBS (e.g., Fiske & Kinder, 1981; Jackson & Marcus, 1975), little work was put into developing a theoretical conceptualization. In Luskin (1990) introduced a systematic attempt, describing PS as a form of political expertise formed through the number and interconnection of thoughts and knowledge related to politics. This is in line with the traditional understanding (Converse, 2006), sticking to the idea of a large belief system that contains political cognitions and constraints. Constraints are defined as beliefs or attitudes with an associative character (Luskin, 1987). They can contain abstract knowledge; for example, categorizing a person as either liberal or conservative, and can have a predictive character by providing an understanding of what political decisions might provoke.

Different conditions of PBS, formed by their size, range and organization of cognitions, lead to different levels of PS. Size refers to the number of cognitions that are embedded. This is a theoretical component since the actual size of a belief system cannot be measured. The range can be either wide or specialized. A person with a specialized PBS has many cognitions concerning a specific topic, for example, climate change, how it affects our planet and mankind, and what accelerates or mitigates climate change. Somebody with a wide PBS has rather facile cognitions regarding all sorts of topics, for example, climate change, economics, social justice, or foreign policy. Lastly, the organization of the held cognitions reflects their interconnection. A high level of organization allows an individual to remember more facts. A sophisticated mind, therefore, consists of a large number of connected cognitions that are part of different categories. In line with previous research, highly politically sophisticated individuals are understood as ideological due to their strong connections between political cognitions and constraints, allowing for stable and integrated attitudes. But even though size, range and organization are key indicators for a sophisticated mind, they are not associated with a specific opinion or political orientation.

Three factors influence how politically sophisticated a PBS is: opportunity, ability and motivation (Luskin, 1990). Opportunity refers to the political information people are exposed to. Having a technical device to read or watch the news, being able to afford a weekly newspaper, or living in a city where political talks can be visited – all this increases one's opportunity to receive information in the first place. Ability is a cognitive aspect, referring to the individual's capacity to retrieve and apply information, usually measured as intelligence. Lastly, motivation describes the drive to use the held information and cognitive ability in forming political judgements, operationalized by a general interest in politics. Luskin's work is probably the most detailed elaboration of PS available.

## POLITICAL SOPHISTICATION AS A PREDICTOR

Over time, the query of political factual knowledge became the common way of measuring PS, as political facts were found to be the best indicator of simple political cognitions (Carpini & Keeter, 1993, 1996). With this pragmatic take on PS, focus shifted from examining the phenomenon to using it to explain other relations. By situating PS in relation to other, more established constructs, the following analysis examines two exemplary areas of research in which PS is employed as a predictor to clarify where the concept reveals theoretical gaps and how these may foster potential misconceptions.

### Values

In research on political values, PS is commonly used to predict how well individuals translate their values – enduring beliefs that guide behaviour and decision‐making (Schwartz, 1992) – into political choices. The central assumption is that individuals, depending on their level of PS, rely on heuristics – cognitive shortcuts that simplify decision‐making under incomplete information (Kahneman et al., 1982; Tversky & Kahneman, 1974) – thereby allowing voters to behave *as if* they were fully informed (Lupia, 1994; Lupia & Mccubbins, 2000). Within this literature, two models are prominent: the General Use Model and the Expertise Interaction Model (also Sophistication Interaction Hypothesis; Sniderman et al., 1991). Both describe how individuals' reliance on heuristics varies with their level of PS when translating values into political decision‐making.

According to the General Use Model, no certain level of PS is necessary to use core values and beliefs to make decisions on social and political issues. People are usually guided by social norms that they have internalized (Feldman, 1988).

These, as well as individual preferences, guide anyone's political decision‐making, making political expert knowledge obsolete (Iyengar, 1990; Lau & Redlawsk, 1997). Some research suggests that political sophistication has little influence on how well citizens' voting choices reflect their policy preferences, as both more and less sophisticated individuals generally succeed in selecting parties that align with their views (Dalton, 2021).

According to the Expertise Interaction Model, though, those who lack political knowledge are more likely to be driven by their feelings and affect towards political groups, while the reasoning of more politically sophisticated individuals is more likely to be driven by a wider range of considerations, including ideology (Sniderman et al., 1991). Highly sophisticated people are, therefore, more consistent in translating their core values into political values (Boonen et al., 2017; Carpini & Keeter, 1996; Lau & Redlawsk, 1997; Sniderman et al., 1991), using domain‐specific rather than broad core values in political decision‐making (Zaller, 1992). This is in line with Converse's conceptualization of PS, which assumes that the highly sophisticated hold a wide range of specific knowledge and abstract concepts of their political surroundings in a cognitive belief system and can build interrelations easily. Accordingly, these people do not use broad values as Feldman supposes but hold domain‐specific principles that influence their decision‐making processes (Goren, 2004).

Studies show evidence for both models (Goren, 2001, 2004): It seems as if everybody bases political judgements on core values, but PS strengthens the relationship. It furthermore leads to crystallized attitudes through a deeper insight into political processes. And though the applied heuristics might differ, everybody uses them to compensate for lacking information (Popkin, 1995; Sniderman et al., 1991).

Although examining the coherence between values and political attitudes is important for understanding democratic processes, certain conceptual issues remain open to discussion. This line of research understands ambivalent attitudes within a belief system as defective, just as the traditional approach did (Converse, 2006). However, this assumption neglects the complexity of belief systems as well as of the political sphere. There is just not one correct way in which ideas can be organized (Baldassarri & Goldberg, 2014; Conover & Feldman, 1984), and different values can compete with each other, leading to seemingly ambivalent political attitudes (Zaller, 1992). In the political sphere, there is no such thing as deriving an objectively correct attitude from values. Tolerance of contradiction, not only in arguments with others, but within oneself, is a common concept in political education. It refers to the necessity to endure contradictions (Transfer für Bildung e.V, 2022). Some might even argue that contradiction or antagonism is the centrepiece of the nature of the political (Mouffe, 2015). Integrating contradictory commitments into one's cognitive belief system can therefore be understood not as a deficiency or inconsistency, but as an expression of political complexity. Both Rokeach and Schwartz acknowledge that individuals may hold value orientations that stand in tension with one another – for example, when instrumental values conflict with terminal values (Rokeach, 1973) or when basic values oppose each other within the broader motivational structure (Schwartz, 1992). However, neither author provides an explicit account of how individuals manage or resolve such value conflicts, leaving open the question of how people navigate these internal tensions. Further, the ability to make political decisions aligned with one's values might be important for democratic engagement, but it is insufficient for capturing the full scope of political behaviour.

Interestingly enough, using PS to explain how people form political opinions makes perfect sense when we understand PS as a reflection of how people think about politics. All studies concerning the General Use or Expertise Interaction Model, though, apply a pragmatic approach to political PS, meaning they ask what people know about politics. This conceptualization is too short‐handed and cannot describe the complex process of gaining an understanding of one's political surroundings.

### Bias

Another research area explores how PS can explain the role of bias. Cognitive biases generally reflect different motivational preferences and judgements (Kunda, 1987). In politics, these motivations are typically divided into directional and accuracy goals (Flynn et al., 2017), each influencing decision‐making. Directionally motivated reasoning, the most common approach to processing political information (Taber & Lodge, 2006), involves seeking information aligned with personal preferences, increasing polarization (Stanley et al., 2020). In contrast, accuracy‐motivated reasoning (Flynn et al., 2017) requires greater cognitive effort to consider all available information for the most precise conclusions (Kahan et al., 2017). The Politically Motivated Reasoning Paradigm (Kahan, 2016) illustrates how individuals interpret evidence in ways consistent with their directional biases, often leading to political misperceptions (Flynn et al., 2017). However, factors like the motivation to be a good citizen, scientific curiosity or ambivalence can counteract these biases, and accuracy‐motivated reasoning can be situationally induced (Flynn et al., 2017).

Research on the Political Sophistication Bias (PS Bias) indicates that, specifically, highly politically sophisticated individuals are likely to indulge in motivated reasoning, resulting in politically polarized attitudes and misperceptions (Bakker et al., 2021; Taber & Lodge, 2006; Vitriol et al., 2023). They show a bias for congruent arguments, spend more time reading contrary arguments and generate arguments as well as denigrating thoughts in response. This is called the paradox of political knowledge: highly politically sophisticated individuals are better at processing information so that it is consistent with their political views (Vegetti & Mancosu, 2020), leading to biased political opinions.

If we understand PS as an advanced way of how people think about the world, it appears counterintuitive that, specifically, highly sophisticated people are prone to the usage of bias. Studies on PS Bias are usually based on a pragmatic conceptualization of PS, measuring factual political knowledge. Therefore, it is not the way we think about the world, but the content of what we know that contributes to biased judgements, though some scholars suggest that political knowledge does not preclude the use of partisan heuristics (Albright, 2009), instead, motivated reasoning seems to be a default mode that is influenced by situational factors (Bayes et al., 2020; Lodge & Taber, 2013; Tappin et al., 2021). A more complex measurement of PS might lead to different results, considering that individual characteristics like the motivation to be a good citizen, science curiosity, and being ambivalent can support accurate reasoning (Flynn et al., 2017), decreasing partisan polarization.

Rather than through PS, the usage of directional‐motivated reasoning might be better understood by applying partisanship research (Bolsen et al., 2014; Leeper & Slothuus, 2014). Van Bavel and Pereira (2018) describe how the “Partisan Brain” influences attitudes, judgements, and behaviours based on political identity strength. People who engage in partisan‐motivated reasoning are more likely to believe false information if it aligns with their preferences. Party affiliation serves as an anchor and thus undermines accuracy goals, leading people to lean toward directional motivated reasoning based on which party they identify with. Partisanship, therefore, serves as a cognitive orientation, simplifying political decisions by directional reasoning based on the attitudes of one's party.

Another limitation of the PS Bias research goes back to the context in which studies are conducted. Most focus on the US with its extremely polarized political landscape, leading to a significant simplification of political decision‐making, which may favour the use of cognitive shortcuts that are sufficient in this context (Boonen et al., 2017; Graber, 2001; Popkin, 1995; Sniderman, 2000). This may lead to a distortion in the observed relationship between PS, political knowledge and bias. Research should therefore examine the PS Bias in less polarized political environments with multiple parties.

To sum this up, the PS Bias goes back to the relationship between political knowledge and polarized political opinions, and might be better explained through Partisan Brain Theory (Van Bavel & Pereira, 2018). Additionally, a theoretical conceptualization of PS as *how* people think about politics might lead to different results.

## MEASURING POLITICAL SOPHISTICATION

As mentioned above, PS research shifted from focusing on how people think about politics to what people know about politics. Though usually referring to its cognitive beginnings (Converse, 2006; Luskin, 1990), studies differ in understanding PS as either a cognitive or pragmatic concept (Gallina, 2023). The cognitive concept focuses on how information is processed and connected, and how political information leads to political attitudes. In short, the cognitive approach focuses on how people think about politics (e.g., Guo & Moy, 1998; Lupton et al., 2015; Luskin, 1990). The pragmatic concept, on the other hand, focuses on what people know about politics as an expression of political expertise (Carpini & Keeter, 1993; Carriere et al., 2019; Zingher & Flynn, 2019). But even though the theoretical foundation of PS differs, it is often operationalized by asking for political knowledge. Since research lacks common ground in theoretical conceptualization as well as in the measurement of PS, Table 1 provides an overview of different studies, their theoretical conceptualization of PS as either pragmatic or cognitive,^1^ as well as their operationalization. The column “Covered Dimensions of PS” in Table 1 refers to different aspects of the concept that are supposed to depict the complexity of the concept. The last column shows how these aspects are operationalized. Though terms like political knowledge, political information, awareness, expertise and sophistication are used interchangeably (Vasilopoulos, 2012), we only included studies that explicitly talked about PS. The table presents a selection of studies that explicitly address PS, aiming to illustrate the diversity of measurement approaches over time. It is not exhaustive but includes representative studies from different decades to highlight key developments and influential methods.

**TABLE 1:** Measuring political sophistication.

Authors	Theoretical concept of PS	Covered dimensions of PS	Operationalization
Converse (2006)	Cognitive	Conceptualization of abstract categories	Open‐ended evaluation of parties Arranging politicians and parties in correct order the liberal‐conservative continuum
Luskin (1990)	Cognitive	Conceptualization of abstract categories	Open‐ended evaluation of parties and politicians (1 Item) Arranging politicians and parties in correct order the liberal‐conservative continuum (1 Item)
		2 Political knowledge	Correctly locate parties on policy issues (11 Items)
Carpini & Keeter (1993)	Pragmatic	Political knowledge	Factual knowledge questions regarding the political system (4 Items) Arranging parties in correct order the liberal‐conservative continuum (1 Item)
Macdonald et al. (1995)^a^	None	Level of education	Educational attainment (1 Item)
		2 Political interest	Interest in political campaign or politics in general (1 Item)
Guo & Moy (1998)	Cognitive	Political interest	Interest in National politics (1 Item)
		2 Political knowledge	Recalling names of political Figures (4 Items)
		3 Cognitive elaboration	Open‐ended questions that asked for causes and solutions for crime problems (2 Items)
		4 Information processing strategies	Questions about how people deal with political information (3 Items)
Gastil & Dillard (1999)	Pragmatic	Attitude coherence	Comparing correlations among attitudes between ideologically similar/dissimilar scales
		2 Attitudinal uncertainty	Number of “not sure” answers
Gomez & Wilson (2001)^a^	Cognitive	Political knowledge	Factual knowledge questions regarding politicians and the political system (8 Items)
Goren (2004)	Cognitive	Political knowledge	Correctly locate parties on policy issues (15 Items)
Highton (2009)^a^	Cognitive	Political knowledge	Factual knowledge questions regarding the political system (5 Items)
		2 Conceptualization of abstract categories	Arranging parties in correct order the liberal‐conservative continuum
Arceneaux et al. (2012)^a^	Cognitive	Level of education	Educational Attainment (1 Item)
		2 Political interest	Interest in politics and public affairs (1 Item)
		3 Political knowledge	Factual knowledge questions regarding the political system (5 Items)
		4 Ideological constraint	Ideological consistency measured across a wide range of political attitudes
Vasilopoulos (2012)	Cognitive	Political knowledge	Factual knowledge questions regarding politicians and the political system (23 Items)
Lupton et al. (2015)^a^	Cognitive	Political interest	Interest in political campaign (1 Item)
		2 Political involvement	Participation in campaign related activities (1 Item)
		3 Political knowledge	External Assessment
Turper & Aarts (2017)^a^	None	Level education	Educational Attainment (1 Item)
		2 Political interest	General interest in politics (1 Item)
Carriere et al. (2019)^a^	Pragmatic	Political knowledge	Factual knowledge questions regarding the political system (3 Items)
Zingher & Flynn (2019)^a^	Pragmatic	Political knowledge	Factual knowledge questions regarding the political system External Assessment
		2 Conceptualization of abstract categories	Arranging politicians and parties in correct order the liberal‐conservative continuum
Vegetti & Mancosu (2020)^a^	Cognitive	Political knowledge	Open factual knowledge questions regarding the political system
Vitriol et al. (2023)	Cognitive	Cognitive reflection test	Logic questions, unrelated to politics (7 Items)
		2 Political knowledge	Factual knowledge questions regarding the political system (8 Items)
		3 Political interest	General interest in and centrality of politics (3 Items)

*Note*: Authors marked with ^a^ have used pre‐existing data sets, for example, ANES.

This exemplary overview clearly shows the lack of stringency in this field of research. Notice that there is no standardized measurement for PS. Accordingly, there is also no statistical validation of the scales. Some authors have different concepts of PS but use the same operationalization (e.g., Carpini & Keeter, 1993; Gomez & Wilson, 2001), while others have the same concept but use different operationalizations (e.g., Arceneaux et al., 2012; Highton, 2009). While most authors describe PS as either a pragmatic or cognitive concept in their theory section, two do not give any theoretical background or explanation at all (Macdonald et al., 1995; Turper & Aarts, 2017).

An ambiguous theoretical description of the construct almost inevitably results in an equally fragmented operationalization, which is reflected in the multitude of different subdimensions used by authors to collect PS (Covered Dimensions of PS), with authors failing to explain how they select these dimensions. Even though some mention similar aspects, the actual operationalization differs. Political Knowledge, for example, is either measured by closed (Arceneaux et al., 2012; Carpini & Keeter, 1993; Carriere et al., 2019; Gomez & Wilson, 2001; Highton, 2009; Vasilopoulos, 2012; Vitriol et al., 2023; Zingher & Flynn, 2019) or open (Guo & Moy, 1998; Vegetti & Mancosu, 2020) factual knowledge questions about the political system, by arranging politicians or parties on an ideological scale (Carpini & Keeter, 1993; Goren, 2004; Luskin, 1990) or by external assessment (Lupton et al., 2015; Zingher & Flynn, 2019), denying that the form of knowledge retrieval has a dramatic effect on the result (Gibson et al., 2020). While dimensions like “Conceptualization of abstract categories”, “Attitude Coherence” and “Ideological Constraint” are in line with Converse's (2006) initial exploration, other dimensions like “Cognitive Elaboration”, “Information Processing Strategies” and the “Cognitive Reflection Test” seek an attempt that focuses more on the logical processing of information. The frequently used dimension “political interest” was originally conceived by Luskin (1990) as a predictor for PS, and “political education” can also be understood as such, at most as a proxy variable, whereby some studies restrict the assumed positive correlation (Highton, 2009; Luskin, 1990). Overall, the findings present a rather heterogeneous picture of the measurement instruments, which may, among other factors, be attributable to the frequent reliance on secondary analyses of existing data sets in many studies (e.g., Macdonald et al., 1995; Zingher & Flynn, 2019).

Apart from the lack of stringent implementation, the query of political knowledge entails several problems. First, it brings up the question of what knowledge we even expect from citizens. What do we consider the average level of knowledge? Second, most items for measuring PS ask who holds which office or ask respondents to classify politicians and parties on a liberal‐conservative continuum. With this operationalization, PS is largely recalling information, neglecting the influence of other aspects like interest and motivation (Flynn et al., 2017; Goren et al., 2022; Highton, 2009). Third, the specific content of the items differs between surveys and quickly loses actuality as well as makes comparisons between different countries difficult. Fourth, some studies suggest that factual knowledge is an unsuitable predictor of political competence (Lupia & McCubbins, 1999, 2000; Popkin, 1995). With the Internet providing constant access to information, the ability to assess its validity is increasingly essential. Fifth, even those holding lots of political knowledge call upon personal experiences when arguing in political debates (Cramer & Toff, 2017). Additionally, scholars usually do not clarify why or how they chose the item content (Barabas et al., 2014; Krosnick et al., 2010). While political knowledge might be a pragmatic predictor in certain scenarios, it is unable to depict the complexity of what it means to be politically sophisticated in a multifaceted political system.

To gain a better understanding of what PS could mean in terms of an individual's role in a democratic society, it is worth taking a look at political education. Representatives of this field emphasize that it is not enough to understand political education as a means of solving a knowledge deficit (Haarmann et al., 2020). Instead, it is about turning people into self‐determined and critically reflective political subjects, forming their opinion beyond their partisan affiliation. This includes teaching central values such as human dignity, justice, equality, solidarity, peace and freedom. Politically educated citizens should be equipped to navigate and address social conflicts. This requires a willingness and ability to engage in discussions as well as tolerance for ambiguity. These aspects fall short in operationalizations of PS as factual knowledge. Understanding PS as a more complex construction than factual knowledge provides the opportunity to better classify previous findings in this area. Accordingly, advancing this field requires both a systematically articulated theoretical framework and a precise operationalization of the construct, enabling clearer differentiation and empirical investigation.

### Summing up the criticism

The above‐mentioned historical development of the construct PS from a complex cognitive construct to the query of political facts shows clear gaps in definition, conceptualization and operationalization. Research that deals with translating values into political attitudes in interaction with PS (Goren, 2004) assumes that a lack of coherence implicates low PS. However, this is too short‐sighted in a political world as complex as ours. There is no singular correct translation of values into attitudes, and values compete with each other. Contradictions arise in political objects themselves and groups (Lipset, 1959; Mouffe, 2015; Transfer für Bildung e.V, 2022). Enduring and reconciling these is not a shortcoming, but an important part of political discussions.

The research on PS and Bias shows how the gaps in PS theory lead to misinterpretations. While PS Bias research claims that those who are highly sophisticated are specifically biased (Bakker et al., 2021), the effect goes back to those who hold higher amounts of political factual knowledge. Therefore, all that these studies can provide is that this bias is not a consequence of how we think about our political surroundings (cognitive paradigm of PS) (Gallina, 2023) but what we know about our political surroundings (pragmatic paradigm of PS) (Gallina, 2023). Future research should inspect whether the PS Bias can be explained through other existing theories, for example, the Partisan Brain approach (Van Bavel & Pereira, 2018).

Last but not least, the lack of consistent measurement reflects the lack of theoretical consistency and makes it nearly impossible to compare the results of different studies regarding PS (see Table 1).

A new approach to PS should not focus exclusively on a specific group of people (e.g., higher education, politicians) but should instead depict how individuals fundamentally absorb and categorize political information in order to form political opinions. Contradictions in individuals' political belief systems must not necessarily be understood as logical errors and thus as a low degree of PS. A look at the actual meaning of the term sophistication provides an adequate starting point for further theoretical assumptions. Sophistication is defined as “the quality of having an understanding of the world and its ways, and having an understanding of the way people behave” (Cambridge Dictionary, n.d.). Transferred into political contexts, this results in having an understanding of the political world, how politics is made, how discussions are held, and how joint conflicts are negotiated.

## RETHINKING POLITICAL SOPHISTICATION IN DEMOCRATIC CONTEXTS

### Contextualizing the political sphere

The criticism presented so far might mislead readers into assuming that PS is a flawed construct with limited added value in understanding political behaviour. However, with some revision, it has great potential. We argue that political sophistication represents a meaningful individual‐level indicator of how people cognitively engage with their political environment. Assuming that democracy relies on the active participation of critically reflective citizens, political sophistication may offer a valuable predictor for understanding political behaviour and decision‐making, which leads to our central innovation: contextualizing PS through understanding it as a citizenship competence in democratic contexts. While most research on PS is situated within democratic contexts, this framing is a novum. This expansion helps to specify what demands individuals need to meet to be considered politically sophisticated, as it provides a contextual reference grounded in the active participation of critically reflective political subjects. Specifically in times of increasing political polarization, where identity politics shapes opinion formation along the lines of (political) group identities, it becomes crucial to understand what supports a return to deliberative political debates (see also Van Zomeren et al., 2024). Democratic systems depend on the exchange of divergent viewpoints, on recognizing and engaging with plural lived realities. In contrast, the dynamics of (affective) polarization tend to oversimplify complex political issues and deepen societal divisions that appear increasingly irreconcilable. We aim to situate the concept of democratic sophistication within a broader theoretical framework in which it is juxtaposed with the dynamics of the Partisan Brain, thereby clarifying how deliberative forms of political engagement stand in contrast to the more dogmatic patterns of behaviour that arise from partisan identity processes. Refining the construct as democratic sophistication and focusing on individual‐level participation in a democratic system, therefore, contributes to greater conceptual clarity while also highlighting its normative and societal relevance.

Even though the context of democracy already provides a certain framework, it is still unclear what the political aspect is to which political sophistication refers. Cognitive approaches to PS typically define the political in narrow terms; by testing respondents' knowledge of specific officeholders (e.g., who occupies which post), they confine *the political* to the system's institutional organization and party leadership. Even though states are important political entities, equating the political sphere solely with the state system is insufficient and neglects other spheres of political organization and participation. Building on Sutor's (1984) conception of politics as the “intentional, joint management of interpersonal situations” (p. 62) and Dewey's (2011) emphasis on the dynamic and interactive character of social life, we adopt a deliberately broad understanding of what constitutes the political. To advance conceptual clarity, we further integrate Arendt and Canovan (2006) view of politics as grounded in human plurality and enacted through the public negotiation of shared concerns, as well as Easton's (1971) definition of politics as the authoritative allocation of values within a society. Synthesizing these perspectives, we conceive of politics as a set of intentional and collectively engaged processes through which matters of shared relevance are negotiated among actors with potentially divergent interests. Such processes are structured by the exercise and contestation of power and aim – implicitly or explicitly – at producing collectively binding decisions or social arrangements. This broad conceptualization situates ordinary citizens, not only institutional elites, as agents capable of political participation and influence, contrary to earlier approaches on PS (e.g. Converse, 2006).

**FIGURE 1:**
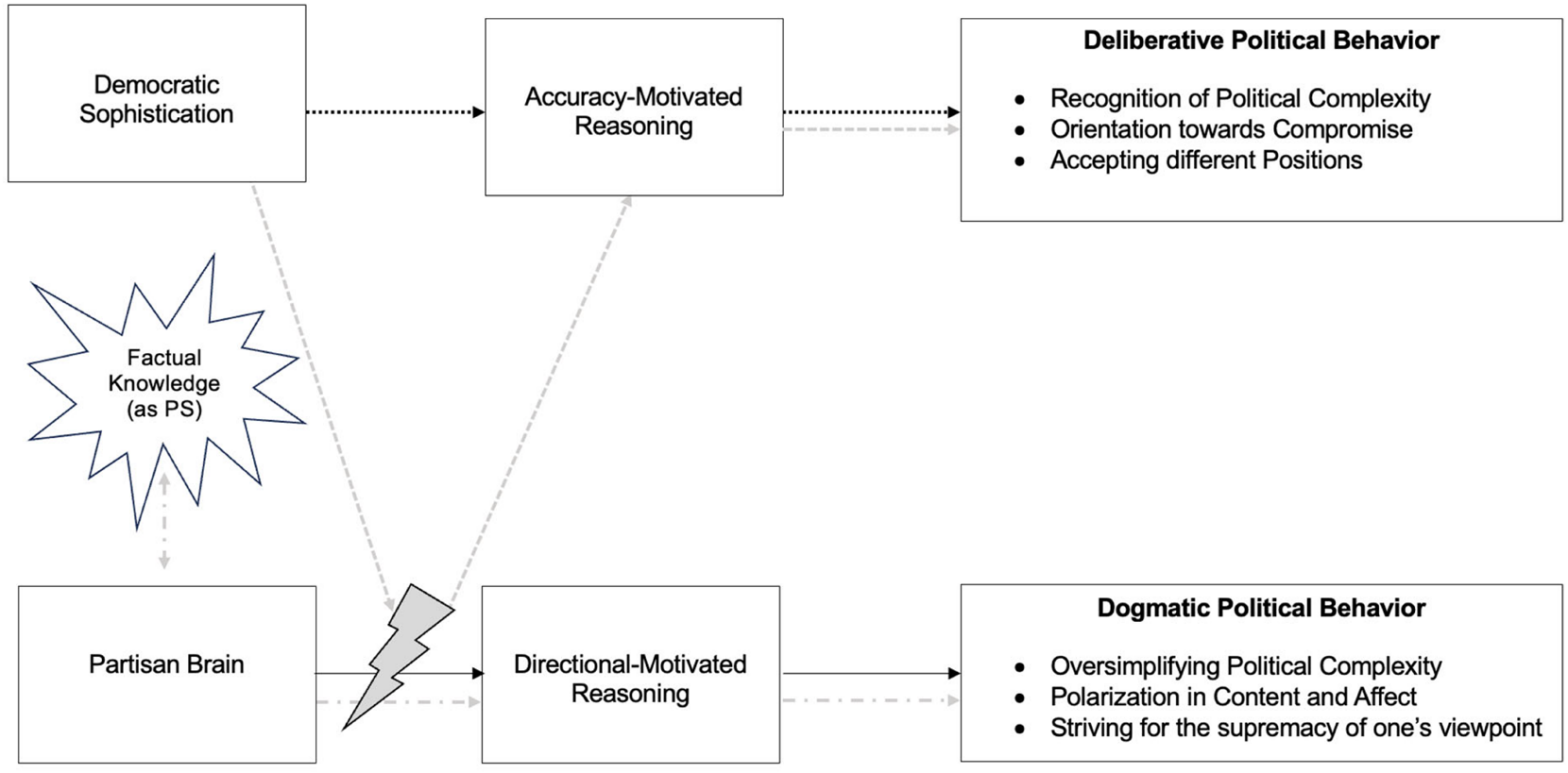
Potential of democratic sophistication. PS, political sophistication.

Although we consider this contextualization and reorientation of the concept to be both promising and important, it is only the first step. Much work remains to be done to create a rigorous theoretical model with clear definitions and implications (Glöckner & Fiedler, 2024), from which a clear operationalization can ultimately emerge. We hope that this framework model will provide initial impetus for the future direction of research on political, or rather, democratic sophistication. We are convinced that this concept offers substantial interdisciplinary value to social psychology, political science and educational scholarship. It has the potential to advance our capacity to predict and explain individuals' political behaviour and decision‐making processes, to improve the design of political education, and to foster more constructive societal dialogue by helping to bridge political polarization. Figure 1, therefore, gives an outlook on the position, or rather function, democratic sophistication could take in the social psychological research of political discussions.

As outlined above, democracy provides the contextual foundation for the reconceptualization of democratic sophistication. Accordingly, Figure 1 distinguishes between two developmental trajectories – one conducive to, and the other detrimental to, the flourishing of democratic coexistence. The upper pathway illustrates mechanisms that are presumed to strengthen the functioning of democratic communities, whereas the lower pathway depicts those that may undermine them. The figure conceptualizes social‐psychological mechanisms as the origins of human behaviour on the left, the resulting cognitive processes in the middle, and political behaviour as the outcome on the right. Democratic Sophistication (DS) is the only newly developed variable in this figure; all others are known to research. This illustration depicts the position and potential of DS, understanding it as a sort of antagonist or buffer to the Partisan Brain.

Figure 1 presents two kinds of political behaviour (PB), deliberative and dogmatic. Deliberative PB is intended to capture forms of political behaviour that strive for compromise and foster social cohesion, while simultaneously recognizing and accepting differences and contradictions – acknowledging, not least, the inherent complexity of the social world (Green et al., 2019). It includes, though not inclusively, deliberative discussion, which fosters democratic decision‐making and is the core of democratic exchange (Mendelberg, 2002). They are characterized by openness to differing opinions and an acknowledgment of political complexity, with an emphasis on compromise over confrontation. In contrast, dogmatic PB (Rokeach, 1954) is characterized by polarization and low tolerance for opposing perspectives. Rather than seeking mutual understanding, dogmatic PB prioritizes argumentative victory and the affirmation of one's prior convictions. Such behaviour treats political interaction as a zero‐sum contest, in which the success of one position presupposes the invalidation of another (Boland & Davidai, 2024). By encouraging rigid, adversarial stances and discouraging constructive dialogue, dogmatic PB fosters social division and thereby poses a threat to the effective functioning of democratic processes.

One source of dogmatic PB, according to the Partisan Brain framework (Van Bavel & Pereira, 2018), is strong identification with one's political ingroup, which can hinder meaningful engagement with political arguments and content. Guided by identity‐related processes, partisans often rely on directionally motivated reasoning when forming political attitudes, thereby reinforcing dogmatic PB (as indicated by the black arrows).

DS, as a specification of PS, may function as an opposing force to Partisan Brain Dynamics by promoting accuracy‐motivated rather than directionally motivated reasoning. This may occur, for example, when individuals recognize the legitimacy of diverse viewpoints or perceive political engagement as a civic responsibility (Flynn et al., 2017). As a result, DS fosters deliberative PB, which is essential for democratic functioning (as indicated by the black dotted arrows).

Previous research has often conceptualized PS as factual political knowledge (e.g., Carriere et al., 2019) and depicted content polarization as a consequence of being highly politically sophisticated (e.g. Taber & Lodge, 2006). However, as noted earlier, high political knowledge is an insufficient operationalization and does not preclude the use of partisan heuristics (Albright, 2009). The PS Bias might therefore actually be explained through Partisan Brain research: partisanship may increase political interest, prompting individuals to selectively attend to congruent information (Peterson & Iyengar, 2021). This, in turn, can foster domain‐specific political knowledge that reinforces one's partisan position. Thus, factual knowledge and partisan brain processes may be reciprocally related, jointly promoting directionally motivated reasoning and, ultimately, dogmatic PB (depicted by grey dot‐dashed arrows). We argue that this dynamic has previously been misinterpreted as the PS Bias and warrants closer examination in future research. In contrast, DS refers to the ability or motivation, regardless of content, that enables individuals to engage critically with political information, buffering the influence of partisanship that drives directionally motivated reasoning (depicted by grey dashed arrows and grey flash), thereby supporting the conditions necessary for deliberative PB (depicted by grey dashed and black dotted arrows).

Figure 1 illustrates how DS could serve as a useful lens for understanding discussion behaviour in democratic systems. While the concept currently lacks a clear conceptualization and operationalization, the criticisms directed at PS provide valuable starting points for further theoretical development. Future research should investigate the social‐psychological foundations that lead individuals to engage in accuracy‐oriented reasoning, which in turn fosters deliberative PB. A revised conceptualization of DS should embrace a broad understanding of what constitutes the political and acknowledge the complexity of contemporary society. It should not be tied to any specific ideology, and political contradictions should be viewed as inherent to democratic life rather than as cognitive or ideological flaws. Further research is needed to refine and validate this construct, as it holds promise for mitigating political polarization and supporting democratic compromise. DS may therefore be of interdisciplinary relevance to political science, social psychology and educational research.

## CONCLUSION

In this paper, we interrogated the conceptual foundations of PS, examined its measurement inconsistencies and proposed a revised framework more attuned to democratic and social‐psychological concerns. Drawing on insights from political science, psychology and educational research, the analysis has highlighted the fragmented and often contradictory ways in which PS has been theorized and operationalized. While PS has historically been associated with individuals' cognitive engagement with political information (Converse, 2006), typically measured through factual political knowledge (Vegetti & Mancosu, 2020), this narrow understanding fails to account for how political knowledge is shaped by value systems, identity processes and contextual factors. The formulation of the PS Bias, suggesting that higher PS is associated with increased susceptibility to motivated reasoning (Vitriol et al., 2023), underscores the need to revisit foundational assumptions about the role of knowledge, motivation and reasoning in political cognition.

In response to these conceptual shortcomings, this paper introduces DS as a more focused and socially relevant reformulation of PS. Rather than emphasizing factual knowledge alone, DS is concerned with how individuals understand, navigate and engage with the political world in ways that are reflective, inclusive and conducive to democratic norms. It builds upon the recognition that democratic systems require not merely informed citizens, but ones who can tolerate complexity, entertain competing perspectives and engage in deliberative forms of political discourse. This reconceptualization moves beyond cognitive competence to include attitudinal openness and a normative commitment to pluralism.

The framework proposed here illustrates how DS may function as a psychological buffer against the polarizing effects of directionally motivated reasoning. Drawing on the Partisan Brain model (Van Bavel & Pereira, 2018), the paper suggests that political knowledge alone does not immunize individuals against bias (Albright, 2009); in fact, it can exacerbate partisan‐driven distortions when reasoning serves identity‐protective functions. DS, by contrast, is hypothesized to promote accuracy‐motivated reasoning, fostering the kind of deliberative political discussions that are essential to democratic life. Figure 1 underscores this dynamic, mapping how DS may shape discussion behaviour via cognitive and motivational mechanisms.

This conceptual pivot from PS to DS has several important implications. First, it reorients scholarly attention towards the democratic function of political reasoning, anchoring PS more firmly in the normative demands of democratic life. Second, it underscores the importance of motivational and contextual factors, which are often under‐theorized in studies of political cognition. Third, it invites interdisciplinary collaboration by bridging psychological insights about identity, motivation and reasoning with political science theories of deliberation and democratic participation. As such, DS may prove particularly fruitful for research in political psychology, where understanding the micro‐dynamics of political discourse is increasingly central to addressing large‐scale challenges like polarization and democratic backsliding.

Of course, this proposal remains preliminary. DS is not yet a fully validated construct, and future research must take up the task of refining its conceptual contours and developing robust measurement strategies. This includes empirical studies that test its predictive validity, distinguish it from related constructs such as open‐mindedness or cognitive complexity, and examine its development across different sociopolitical contexts. Moreover, research must critically assess the normative assumptions embedded in the concept and remain sensitive to how DS may manifest differently across cultural and institutional settings.

In sum, Democratic Sophistication offers a promising new lens through which to examine the psychological underpinnings of political engagement. By emphasizing the capacity for reflective, inclusive and complexity‐sensitive reasoning, DS reframes PS not merely as an index of political knowledge, but as a civic capacity vital to the health of democratic systems. Reinvigorating this line of inquiry may help scholars and practitioners alike better understand how individuals navigate a complex and often polarized political world – and, crucially, how they might do so in ways that sustain, rather than undermine, democratic life.

## AUTHOR CONTRIBUTIONS

**Klara Steinmetz:** Conceptualization; writing – original draft; visualization; project administration. **Frank Asbrock:** Writing – review and editing; supervision.

## CONFLICT OF INTEREST STATEMENT

The authors report no conflicts of interest.

## Data Availability

Data sharing not applicable to this article as no datasets were generated or analyzed during the current study.

## Footnotes

^1^
We have classified the approaches as either pragmatic or cognitive, depending on how the authors described PS in their theory. That is important, since authors sometimes refer to the same literature, but emphasize different aspects. We have categorized as ‘cognitive concept’ if PS was understood as more than just a high amount of political knowledge.

## References

[bjso70035-bib-0001] Albright, J. J. (2009). Does political knowledge erode party attachments?: A review of the cognitive mobilization thesis. Electoral Studies, 28(2), 248–260. 10.1016/j.electstud.2009.01.001

[bjso70035-bib-0002] Arceneaux, K. , Johnson, M. , & Maes, H. H. (2012). The genetic basis of political sophistication. Twin Research and Human Genetics, 15(1), 34–41. 10.1375/twin.15.1.34 22784451

[bjso70035-bib-0003] Arendt, H. , & Canovan, M. (2006). The human condition (2nd ed.). University of Chicago Press.

[bjso70035-bib-0004] Bakker, B. N. , Lelkes, Y. , & Malka, A. (2021). Reconsidering the link between self‐reported personality traits and political preferences. American Political Science Review, 115(4), 1482–1498. 10.1017/S0003055421000605

[bjso70035-bib-0005] Baldassarri, D. , & Goldberg, A. (2014). Neither ideologues nor agnostics: Alternative voters' belief system in an age of partisan politics. American Journal of Sociology, 120(1), 45–95. 10.1086/676042 25705780

[bjso70035-bib-0006] Ball, T. , Dagger, R. , & O'Neill, D. I. (2014). Political ideologies and the democratic ideal (9th ed.). Pearson.

[bjso70035-bib-0007] Barabas, J. , Jerit, J. , Pollock, W. , & Rainey, C. (2014). The question(s) of political knowledge. American Political Science Review, 108(4), 840–855. 10.1017/S0003055414000392

[bjso70035-bib-0008] Barton, A. H. , & Parsons, R. W. (1977). Measuring belief system structure. Public Opinion Quarterly, 41(2), 159. 10.1086/268372

[bjso70035-bib-0009] Bayes, R. , Druckman, J. N. , Goods, A. , & Molden, D. C. (2020). When and How Different Motives Can Drive Motivated Political Reasoning. Political Psychology, 41(5), 1031–1052. 10.1111/pops.12663

[bjso70035-bib-0010] Beierlein, C. , Asbrock, F. , Kauff, M. , & Schmidt, P. (2015). Kurzskala Autoritarismus (KSA‐3). Zusammenstellung sozialwissenschaftlicher Items und Skalen (ZIS). 10.6102/ZIS228

[bjso70035-bib-0011] Berelson, B. R. , Lazarsfeld, P. F. , & McPhee, W. N. (1955). Voting, a study of opinion formation in a presidential campaign. Journal of Marketing, 20(2), 202. 10.2307/1247297

[bjso70035-bib-0012] Boland, F. K. , & Davidai, S. (2024). Zero‐sum beliefs and the avoidance of political conversations. Communications Psychology, 2(1), 43. 10.1038/s44271-024-00095-4 39242849 PMC11332089

[bjso70035-bib-0013] Bolsen, T. , Druckman, J. N. , & Cook, F. L. (2014). The influence of partisan motivated reasoning on public opinion. Political Behavior, 36(2), 235–262. 10.1007/s11109-013-9238-0

[bjso70035-bib-0014] Bonikowski, B. , Feinstein, Y. , & Bock, S. (2019). The partisan sorting of ‘America’: How nationalist cleavages shaped the 2016 U.S. *Presidential Election*. SocArXiv. 10.31235/osf.io/pmg95

[bjso70035-bib-0015] Boonen, J. , Pedersen, E. F. , & Hooghe, M. (2017). The effect of political sophistication and party identification on voter–party congruence. A comparative analysis of 30 countries. Journal of Elections, Public Opinion and Parties, 27(3), 311–329. 10.1080/17457289.2016.1273226

[bjso70035-bib-0016] Bovan, K. (2022). How to vote correctly: An experimental study on the impact of political sophistication, cognitive load, and decision‐making strategies. Anali Hrvatskog Politološkog Društva, 19(1), 179–211. 10.20901/an.19.01

[bjso70035-bib-0017] Cambridge Dictionary . (n.d.). Sophistication. Cambridge Dictionary. https://dictionary.cambridge.org/dictionary/english/sophistication

[bjso70035-bib-0018] Campbell, A. , Converse, P. E. , Miller, W. E. , & Stokes, D. E. (1960). The American Voter. John Wiley.

[bjso70035-bib-0019] Carpini, M. X. , & Keeter, S. (1996). What Americans know about politics and why it matters. Yale University Press.

[bjso70035-bib-0020] Carpini, M. X. D. , & Keeter, S. (1993). Measuring Political Knowledge: Putting First Things First. American Journal of Political Science, 37(4), 1179. 10.2307/2111549

[bjso70035-bib-0021] Carriere, K. R. , Hendricks, M. J. , & Moghaddam, F. M. (2019). Sophisticated but scared: The effects of political sophistication, right‐wing authoritarianism, and threat on civil liberty restrictions. Analyses of Social Issues and Public Policy, 19(1), 256–281. 10.1111/asap.12186

[bjso70035-bib-0022] Conover, P. J. , & Feldman, S. (1984). Group identification, values, and the nature of political beliefs. American Politics Quarterly, 12(2), 151–175. 10.1177/1532673X8401200202

[bjso70035-bib-0023] Converse, P. E. (2006). The nature of belief systems in mass publics (1964). Critical Review, 18(1–3), 1–74. 10.1080/08913810608443650

[bjso70035-bib-0024] Cramer, K. J. , & Toff, B. (2017). The fact of experience: Rethinking political knowledge and civic competence. Perspectives on Politics, 15(3), 754–770. 10.1017/S1537592717000949

[bjso70035-bib-0025] Dalton, R. (2021). The representation gap and political sophistication: A contrarian perspective. Comparative Political Studies, 54(5), 889–917. 10.1177/0010414020957673

[bjso70035-bib-0026] Dassonneville, R. , & Dejaeghere, Y. (2014). Bridging the ideological space: A cross‐national analysis of the distance of party switching. European Journal of Political Research, 53(3), 580–599. 10.1111/1475-6765.12049

[bjso70035-bib-0027] Dewey, J. (2011). The public and its problems (Nachdruck). Swallow Press.

[bjso70035-bib-0028] Downs, A. (1957). An economic theory of democracy.

[bjso70035-bib-0029] Duckitt, J. , & Sibley, C. G. (2016). The dual process motivational model of ideology and prejudice. In C. G. Sibley & F. K. Barlow (Eds.), The Cambridge handbook of the psychology of prejudice (1st ed., pp. 188–221). Cambridge University Press. 10.1017/9781316161579.009

[bjso70035-bib-0030] Easton, D. (1971). The political system: An inquiry into the state of political science (2nd ed.). Knopf.

[bjso70035-bib-0031] Feldman, S. (1988). Structure and consistency in public opinion: The role of core beliefs and values. American Journal of Political Science, 32(2), 416. 10.2307/2111130

[bjso70035-bib-0032] Field, J. , & Anderson, R. (1969). Ideology in the public's conceptualization of the 1964 election. Public Opinion Quarterly, 33(3), 380–398.

[bjso70035-bib-0033] Fiske, S. T. , & Kinder, D. R. (1981). Ement, expertise, and schema use: Evidence from political cognition. In N. Cantor & J. F. Khilstrom (Eds.), Personality, cognition, and social interaction (pp. 171–190). Lawrence Erlbaum.

[bjso70035-bib-0034] Flynn, D. J. , Nyhan, B. , & Reifler, J. (2017). The nature and origins of misperceptions: Understanding false and unsupported beliefs about politics. Political Psychology, 38(S1), 127–150. 10.1111/pops.12394

[bjso70035-bib-0035] Gallina, M. (2023). The concept of political sophistication: Labeling the unlabeled. Political Studies Review, 21(4), 836–846. 10.1177/14789299221146058

[bjso70035-bib-0150] Gastil, J. , & Dillard, J. P. (1999). Increasing political sophistication through public deliberation. Political Communication, 16(1), 3–23. 10.1080/105846099198749

[bjso70035-bib-0036] Gibson, J. , Claassen, C. , & Barceló, J. (2020). Deplorables: Emotions, political sophistication, and political intolerance. American Politics Research, 48(2), 252–262. 10.1177/1532673X18820864

[bjso70035-bib-0037] Glöckner, A. , & Fiedler, S. (2024). Theory specification and theory building in psychology. Zeitschrift für Psychologie, 232(1), 68–70. 10.1027/2151-2604/a000541

[bjso70035-bib-0038] Gomez, B. T. , & Wilson, J. M. (2001). Political sophistication and economic voting in the American electorate: A theory of heterogeneous attribution. American Journal of Political Science, 45(4), 899. 10.2307/2669331

[bjso70035-bib-0039] Goren, P. (2001). Core principles and policy reasoning in mass publics: A test of two theories. British Journal of Political Science, 31, 159–177. 10.1017/S0007123401000072

[bjso70035-bib-0040] Goren, P. (2004). Political sophistication and policy reasoning: A reconsideration. American Journal of Political Science, 48(3), 462–478. 10.1111/j.0092-5853.2004.00081.x

[bjso70035-bib-0041] Goren, P. , Smith, B. , & Motta, M. (2022). Correction to: Human values and sophistication interaction theory. Political Behavior, 44(2), 1053. 10.1007/s11109-022-09785-3

[bjso70035-bib-0042] Graber, D. A. (2001). Processing politics: Learning from television in the internet age. University of Chicago Press.

[bjso70035-bib-0043] Green, J. , Kingzette, J. , & Neblo, M. (2019). Deliberative democracy and political decision making. In J. Green , J. Kingzette , & M. Neblo (Eds.), Oxford research encyclopedia of politics. Oxford University Press. 10.1093/acrefore/9780190228637.013.917

[bjso70035-bib-0044] Guo, Z. , & Moy, P. (1998). Medium or message? Predicting dimensions of political sophistication. International Journal of Public Opinion Research, 10(1), 25–50. 10.1093/ijpor/10.1.25

[bjso70035-bib-0045] Haarmann, M. P. , Kenner, S. , & Lange, D. (2020). Demokratie, demokratisierung und das demokratische. Aufgaben und zugänge der politischen bildung. Eine hinführung. In M. P. Haarmann , S. Kenner , & D. Lange (Eds.), Demokratie, demokratisierung und das demokratische (pp. 1–6). Springer.

[bjso70035-bib-0046] Highton, B. (2009). Revisiting the relationship between educational attainment and political sophistication. The Journal of Politics, 71(4), 1564–1576. 10.1017/S0022381609990077

[bjso70035-bib-0047] Hyman, H. H. , & Sheatsley, P. B. (1947). Some reasons why information campaigns fail. Public Opinion Quarterly, 11(3), 412. 10.1086/265867

[bjso70035-bib-0048] Iyengar, S. (1990). Framing responsibility for political issues: The case of poverty. Political Behavior, 12(1), 19–40. 10.1007/BF00992330

[bjso70035-bib-0049] Jackson, T. H. , & Marcus, G. E. (1975). Political competence and ideological constraint. Social Science Research, 4(2), 93–111. 10.1016/0049-089X(75)90006-X

[bjso70035-bib-0050] Jost, J. T. , Federico, C. M. , & Napier, J. L. (2009). Political ideology: Its structure, functions, and elective affinities. Annual Review of Psychology, 60(1), 307–337. 10.1146/annurev.psych.60.110707.163600 19035826

[bjso70035-bib-0051] Kahan, D. M. (2016). The politically motivated reasoning paradigm, Part 1: What politically motivated reasoning is and how to measure it. In R. A. Scott & S. M. Kosslyn (Eds.), Emerging Trends in Social and Behavioral Sciences. 10.1002/9781118900772.etrds0417

[bjso70035-bib-0052] Kahan, D. M. , Landrum, A. , Carpenter, K. , Helft, L. , & Hall Jamieson, K. (2017). Science curiosity and political information processing. Political Psychology, 38(S1), 179–199. 10.1111/pops.12396

[bjso70035-bib-0053] Kahneman, D. , Slovic, P. , & Tversky, A. (1982). Judgment under uncertainty: Heuristics and biases (1st ed.). Cambridge University Press. 10.1017/CBO9780511809477 17835457

[bjso70035-bib-0054] Kölln, A.‐K. (2018). Political sophistication affects how citizens' social policy preferences respond to the economy. West European Politics, 41(1), 196–217. 10.1080/01402382.2017.1332314

[bjso70035-bib-0055] Krosnick, J. A. , Visser, P. S. , & Harder, J. (2010). The psychological underpinnings of political behavior. In S. T. Fiske , D. T. Gilbert , & G. Lindzey (Eds.), Handbook of social psychology (1st ed.). Wiley. 10.1002/9780470561119.socpsy002034

[bjso70035-bib-0056] Kunda, Z. (1987). Motivated inference: Self‐serving generation and evaluation of causal theories. Journal of Personality and Social Psychology, 53(4), 636–647. 10.1037/0022-3514.53.4.636

[bjso70035-bib-0057] Lau, R. (1986). Political schemata, candidate evaluations, and voting behaviour. In A. H. Miller , M. P. Wattenberg , & O. Malanchuk (Eds.), Political cognition (pp. 95–126). Erlbaum.

[bjso70035-bib-0058] Lau, R. R. , & Redlawsk, D. P. (1997). Voting correctly. American Political Science Review, 91(3), 585–598. 10.2307/2952076

[bjso70035-bib-0059] Leeper, T. J. , & Slothuus, R. (2014). Political parties, motivated reasoning, and public opinion formation. Political Psychology, 35(S1), 129–156. 10.1111/pops.12164

[bjso70035-bib-0060] Lippmann, W. (1922). Public opinion. Free Press.

[bjso70035-bib-0061] Lipset, S. M. (1959). Some social requisites of democracy: Economic development and political legitimacy. American Political Science Review, 53(1), 69–105. 10.2307/1951731

[bjso70035-bib-0062] Lodge, M. , & Taber, C. S. (2013). The rationalizing voter (1st ed.). Cambridge University Press. 10.1017/CBO9781139032490

[bjso70035-bib-0063] Lupia, A. (1994). The effect of information on voting behavior and electoral outcomes: An experimental study of direct legislation. Public Choice, 78(1), 65–86. 10.1007/BF01053366

[bjso70035-bib-0064] Lupia, A. , & McCubbins, M. D. (1999). The democratic dilemma: Can citizens learn what they need to know? Cambridge Univ. Press.

[bjso70035-bib-0065] Lupia, A. , & Mccubbins, M. D. (2000). Representation or abdication? How citizens use institutions to help delegation succeed. European Journal of Political Research, 37(3), 291–307. 10.1111/1475-6765.00514

[bjso70035-bib-0066] Lupton, R. N. , Myers, W. M. , & Thornton, J. R. (2015). Political sophistication and the dimensionality of elite and mass attitudes, 1980–2004. The Journal of Politics, 77(2), 368–380. 10.1086/679493

[bjso70035-bib-0067] Luskin, R. C. (1987). Measuring political sophistication. American Journal of Political Science, 31(4), 856. 10.2307/2111227

[bjso70035-bib-0068] Luskin, R. C. (1990). Explaining political sophistication. Political Behavior, 12, 331–361.

[bjso70035-bib-0069] Macdonald, S. E. , Rabinowitz, G. , & Listhaug, O. (1995). Political sophistication and models of issue voting. British Journal of Political Science, 25(4), 453–483. 10.1017/S0007123400007316

[bjso70035-bib-0070] Marcus, G. E. , Neuman, W. R. , & MacKuen, M. (2000). Affective intelligence and political judgment. University of Chicago Press.

[bjso70035-bib-0071] Mendelberg, T. (2002). The deliberative citizen: Theory and evidence. In Political decision making, deliberation and participation (pp. 151–193). Elsevier Science Ltd. https://www.princeton.edu/~talim/mendelberg%20‐%20deliberative%20citizen.pdf

[bjso70035-bib-0072] Milačić, F. (2021). The negative impact of polarization on democracy. FES Democracy of the Future. https://democracy.fes.de/e/the‐negative‐impact‐of‐polarization‐on‐democracy.html

[bjso70035-bib-0073] Mouffe, C. (2015). In O. Marchart (Ed.), Trans.; durchgesehene Nachaufl Das demokratische paradox. Turia + Kant.

[bjso70035-bib-0074] Nie, N. , Verba, S. , & Petrocik, J. (1976). The changing American voter. Havard University Press.

[bjso70035-bib-0075] Pateman, C. (1970). Participation and Democratic Theory (1st ed.). Cambridge University Press. 10.1017/CBO9780511720444

[bjso70035-bib-0076] Peterson, E. , & Iyengar, S. (2021). Partisan gaps in political information and information‐seeking behavior: Motivated reasoning or cheerleading? American Journal of Political Science, 65(1), 133–147. 10.1111/ajps.12535

[bjso70035-bib-0077] Pierce, J. (1970). Party identification and the changing role of ideology in American politics. Midwest Journal of Political Science, 14, 25–42.

[bjso70035-bib-0078] Popkin, S. L. (1995). Information shortcuts and the reasoning voter. In B. N. Grofman (Ed.), Information, participation and choice: An economic theory of democracy in perspective (pp. 17–35). University of Michigan Press.

[bjso70035-bib-0079] Reese, G. , & Jacob, L. (2015). Principles of environmental justice and pro‐environmental action: A two‐step process model of moral anger and responsibility to act. Environmental Science & Policy, 51, 88–94. 10.1016/j.envsci.2015.03.011

[bjso70035-bib-0080] Rokeach, M. (1954). The nature and meaning of dogmatism. Psychological Review, 61(3), 194–204. 10.1037/h0060752 13167246

[bjso70035-bib-0081] Rokeach, M. (1973). The nature of human values. Free Press.

[bjso70035-bib-0082] Schwartz, S. H. (1992). Universals in the content and structure of values: Theoretical advances and empirical tests in 20 countries. Elsevier, 25, 1–65. 10.1016/S0065-2601(08)60281-6

[bjso70035-bib-0083] Sherkat, D. E. (2021). Cognitive sophistication, religion, and the trump vote. Social Science Quarterly, 102(1), 179–197. 10.1111/ssqu.12906

[bjso70035-bib-0084] Sniderman, P. M. (2000). Taking sides: A fixed choice theory of political reasoning. In A. Lupia , M. D. McCubbins , & S. L. Popkin (Eds.), Elements of reason (1st ed., pp. 67–84). Cambridge University Press. 10.1017/CBO9780511805813.004

[bjso70035-bib-0085] Sniderman, P. M. , Brody, R. A. , & Tetlock, P. E. (1991). Reasoning and choice: Explorations in political psychology (1st ed.). Cambridge University Press. 10.1017/CBO9780511720468

[bjso70035-bib-0086] Stanley, M. L. , Henne, P. , Yang, B. W. , & De Brigard, F. (2020). Resistance to position change, motivated reasoning, and polarization. Political Behavior, 42(3), 891–913. 10.1007/s11109-019-09526-z

[bjso70035-bib-0087] Stanovich, K. E. , Toplak, M. E. , & West, R. F. (2019). Intelligence and rationality. In R. J. Sternberg (Ed.), The Cambridge handbook of intelligence (2nd ed., pp. 1106–1139). Cambridge University Press. 10.1017/9781108770422.047

[bjso70035-bib-0088] Sutor, B. (1984). Neue grundlegung politischer bildung. 1: Politikbegriff und politische anthropologie/Bernhard Sutor Schöningh.

[bjso70035-bib-0089] Taber, C. S. , & Lodge, M. (2006). Motivated skepticism in the evaluation of political beliefs. American Journal of Political Science, 50(3), 755–769. 10.1111/j.1540-5907.2006.00214.x

[bjso70035-bib-0090] Tappin, B. M. , Pennycook, G. , & Rand, D. G. (2021). Rethinking the link between cognitive sophistication and politically motivated reasoning. Journal of Experimental Psychology: General, 150(6), 1095–1114. 10.1037/xge0000974 33119359

[bjso70035-bib-0091] Transfer für Bildung e.V . (2022). Widerspruchstoleranz. Profession Politische Bildung. https://profession‐politischebildung.de/grundlagen/grundbegriffe/widerspruchstoleranz/

[bjso70035-bib-0092] Turper, S. , & Aarts, K. (2017). Political trust and sophistication: Taking measurement seriously. Social Indicators Research, 130(1), 415–434. 10.1007/s11205-015-1182-4 28163349 PMC5250644

[bjso70035-bib-0093] Tversky, A. , & Kahneman, D. (1974). Judgment under uncertainty: Heuristics and biases: Biases in judgments reveal some heuristics of thinking under uncertainty. Science, 185(4157), 1124–1131. 10.1126/science.185.4157.1124 17835457

[bjso70035-bib-0094] Van Bavel, J. J. , & Pereira, A. (2018). The partisan brain: An identity‐based model of political belief. Trends in Cognitive Sciences, 22(3), 213–224. 10.1016/j.tics.2018.01.004 29475636

[bjso70035-bib-0095] Van Der Heijden, E. , & Verkuyten, M. (2020). Educational attainment, political sophistication and anti‐immigrant attitudes. Journal of Social and Political Psychology, 8(2), 600–616. 10.5964/jspp.v8i2.1334

[bjso70035-bib-0096] Van Zomeren, M. (2013). Four core social‐psychological motivations to undertake collective action. Social and Personality Psychology Compass, 7(6), 378–388. 10.1111/spc3.12031

[bjso70035-bib-0097] Van Zomeren, M. , d'Amore, C. , Pauls, I. L. , Shuman, E. , & Leal, A. (2024). The intergroup value protection model: A theoretically integrative and dynamic approach to intergroup conflict escalation in democratic societies. Personality and Social Psychology Review, 28(2), 225–248. 10.1177/10888683231192120 37667857 PMC11010547

[bjso70035-bib-0098] Vasilopoulos, P. (2012). Political sophistication: Theoretical, methodological and empirical perspectives [Εθνικό και Καποδιστριακό Πανεπιστήμιο Αθηνών (ΕΚΠΑ), Τμήμα Επικοινωνίας και Μέσων Μαζικής Ενημέρωσης]. 10.12681/eadd/38762

[bjso70035-bib-0099] Vegetti, F. , & Mancosu, M. (2020). The impact of political sophistication and motivated reasoning on misinformation. Political Communication, 37(5), 678–695. 10.1080/10584609.2020.1744778

[bjso70035-bib-0100] Vitriol, J. A. , Sandor, J. , Vidigal, R. , & Farhart, C. (2023). On the independent roles of cognitive & political sophistication: Variation across attitudinal objects. Applied Cognitive Psychology, 37(2), 319–331. 10.1002/acp.4022

[bjso70035-bib-0101] Weissberg, R. (1976). The politics of political socialization. Youth & Society, 8(2), 117–145. 10.1177/0044118X7600800203

[bjso70035-bib-0102] Weitz‐Shapiro, R. , & Winters, M. S. (2017). Can citizens discern? Information credibility, political sophistication, and the punishment of corruption in Brazil. The Journal of Politics, 79(1), 60–74. 10.1086/687287

[bjso70035-bib-0103] Zaller, J. R. (1992). The nature and origins of mass opinion (1st ed.). Cambridge University Press. 10.1017/CBO9780511818691

[bjso70035-bib-0104] Zingher, J. N. , & Flynn, M. E. (2019). Does polarization affect even the inattentive? Assessing the relationship between political sophistication, policy orientations, and elite cues. Electoral Studies, 57, 131–142. 10.1016/j.electstud.2018.11.007

